# Editorial Notes

**Published:** 1920-12

**Authors:** 


### Editorial Notes.

**The Voluntary Hospitals as a Public Trust.**

THE financial crisis that has overtaken the voluntary hospitals may be the most obvious of the difficulties that those who are responsible for their management have to face, yet it is only one of the many problems calling for solution—problems of profound national concern, and demanding deep thinking.

It may be useful to touch on the main questions at issue, and in doing so to emphasise one essential consideration that should dominate all our constructive plans for re-establishing our hospital system on a sound basis, viz. that the voluntary hospitals were built, equipped, and largely endowed by private philanthropy in co-partnership with the medical staffs for the benefit of necessitous patients requiring medical assistance such as the hospitals afford, and for whom no such provision was made by the State or any other public body.

These gifts in kind, together with free medical service for the sick poor not otherwise provided for, constitute a public trust, and the managers of the various hospitals are guardians of that trust.

Now it might appear a relatively simple solution of the whole matter if the hospitals maintain as many beds as their resources allow for those patients who can only afford small contributions towards their maintenance and those who must be admitted free as heretofore, and then fill their remaining beds with patients who can pay the entire cost of maintenance, taking precautions to safeguard their funds, to which the most necessitous have the greatest claim, from being encroached on by those who are better off.

It is, however, to the credit of our voluntary hospital system that the work has been so well carried out and developed that they have become not only the centres for scientific research and education and the great training schools for the medical and nursing professions, but have so developed their resources that the most costly and best equipped nursing homes in some respects fall short of hospitals for patients requiring special treatment.

Briefly, then, the crisis is due to three main factors :—

1. The increasing appreciation of hospitals for treatment by an ever widening section of the community.
2. The increased cost of maintenance, and not the "cost of living" alone, but the cost of providing all the scientific resources that have become an essential feature in every well-equipped hospital.
3. The inability of the charitable public to provide the whole of the funds to meet these heavy and increasing costs.

Realising as we must that the patients who wish to go to hospitals, and for whom an up-to-date hospital service is practically an urgent necessity, are no longer the necessitous poor only, we may consider them under four heads :—

(*a*) Necessitous persons.

(*b*) Persons of only moderate means who can contribute to the cost of maintenance *but cannot afford more.*

(*c*) Patients who are provided for by the State or other public body or by the funds of a "Society."

(*d*) Persons of comfortable means, able to pay both the hospital charges and the fees of the medical attendant, but preferring to have treatment in a hospital rather than in a nursing home or in their own house.

Now the two first groups may be taken to comprise patients for whom the hospitals were built and established, and who have therefore a vested interest therein. To these the honorary medical staffs accord their services free.

The two latter categories comprise those for whom it may, or may not, be expedient on various grounds to open the voluntary hospital doors. Is it not possible to provide for such on special conditions, so that they shall not encroach on the accommodation provided and required for the two first groups? Obviously these two groups (*c*) and (*d*) are patients who are in no sense entitled to the gifts of the private philanthropist or to the honorary services of the medical staff. Public bodies have provided separate institutions for cases of lunacy and infectious disease because they are a danger to the community, and if public services provide for other categories, *e.g.* cases under the Pensions Ministry, the charges for medical services must, of course, be defrayed by the State or other public body. We must not confuse such payments by the State with State aid—instead of the State aiding the hospital, the hospital would aid the State, and must be repaid its outlay on State work. Moreover the patients who are not necessitous must not be allowed to encroach on the accommodation required by those who are.

No regulations by committees or differentiation of the patients into those who can and those who ought not to contribute can be effective without the assistance of trained almoners, otherwise, however worthy of consideration, the really poor patient will soon be squeezed out by the relatively well-to-do.

It would appear essential that the accounts of hospitals should be kept so as to differentiate between those who, on the one hand, pay for themselves or who are paid for by public bodies, and therefore do not involve the hospital in any cost whatever, and those who, on the other hand, are provided for by private philanthropy.

At a recent meeting of the Bristol Pensions Committee for the purpose of urging on the Ministry of Pensions the desirability of making provision for the hospital treatment in Bristol of ex-service men who belong to this city Alderman Sheppard, who presided, suggested the possibility of co-operation between the voluntary hospitals and the Ministry, and said that the various institutions could find the accommodation and the Government must be prepared to pay the whole cost of the treatment required, including payment to the honorary staff. There is a very strong feeling that Bristol ex-service men whose conditions require hospital treatment should no longer be sent away from their homes to neighbouring cities; but it is certain that the funds of the civil hospitals, being unequal to meet the costs of poor civil patients, should not bear any part of the cost of patients for whom the Ministry is pledged to provide. This illustrates only one of the many categories of patients who may be considered as suitable for our hospitals, but for whom special conditions must be clearly laid down if the voluntary hospitals as such are to continue to exist and to carry on their splendid services to the commonwealth.

The Poor Law Infirmaries are, doubtless, destined to play an all-important part in the near future in meeting the national shortage of hospital service. Immense possibilities lie in this direction. Already the Bristol Guardians have made a good start in developing their work and resources. We propose to discuss this question in a subsequent issue.

**An Almoner Department for the Bristol Voluntary Hospitals.**

One of the most urgent and crying needs of the day in connection with the Bristol voluntary hospitals is the institution of a properly-equipped Almoner Department, common to all the hospitals. It would prevent the philanthropic subscriber feeling that perchance his self-denial is misplaced and that his contributions are filched by patients who can quite well afford to provide for their own needs.

An Almoner requires special training in work which is highly responsible, and calls for tact, knowledge of the work, and organising capacity, and when, as in Bristol, there are several hospitals, it is essential that the Almoner's Department of each should be working in collaboration under one head, if reduplication and waste of money and effort is to be avoided. Furthermore, the department should ensure the equal collaboration of the lay committees and the honorary medical staffs of the various institutions. The work of such a department is by no means confined to sifting out patients whose financial status renders them improper persons for treatment in a voluntary hospital, for the inquiry reveals and provides for many cases where there is a call for sympathy and help outside mere treatment. The heads of the department would be cognisant of all the manifold charitable organisations in and around the city, and would constitute an invaluable source of information to many a poor patient, and to many who want to get into touch with those who really need help.

Such a department demands one or more specially-trained and paid heads, clerical assistance, and a number of co-workers. It would save money, economise effort, and give confidence to the private donor and the medical staffs. It seems extraordinary that Bristol has hitherto failed to institute such a department ; but now that patients who can afford to pay even the full cost of maintenance, and now also that those for whom the " public services " provide, are to be considered proper cases for hospitals, such a department is more than ever necessary for the safe-guarding and proper organisation of medical charity.

